# A Gas Furnace for Firing Dental Enamels and Porcelains, Causes of “Gasing” in Firing Teeth

**Published:** 1885-02

**Authors:** William Herbert Rollins


### A Gas Furnace for Firing Dental Enamels and Porcelains, Causes of "Gasing" in Firing Teeth.

BY WILLIAM HERBERT ROLLINS.

The two subjects are combined because by means of the former I have been able to discover the latter.

The furnace here described will give a heat from a dull red to a light so bright that an object in the muffle is invisible. The furnace consists of a cylinder of sheet iron ten inches in diameter and eleven inches high. It has a handle and an iron door. The cover is a similar cylinder two inches deep. Both are lined with porous fire clay two inches thick. The furnace walls have four holes in them. Three of these holes give passage to three tuyeres, the fourth is for a No. 3 muffle, the mouth of which is closed by an iron door lined with asbestos. The door is perforated by a platinum tube, through which passes the platinum trial rod with a small spoon end for the test piece. This rod can be withdrawn from time to time to look at the test piece. At the front of the furnace are two iron tubes, one for gas, the other for air. These pass under the fire-clay arch, where they are brought to a yellow heat by the waste gases as they escape through the slit in the top of the furnace. Each of these tubes divides into three branches at the back of the furnace where the gas tubes enter the air tubes. In this way three double tuyeres are made, their mouths corresponding with the holes in the furnace. This arrangement is necessary, for if the heated air and gas are allowed to mix before reaching the mouths of the tuyeres these would be destroyed by the intense heat. A similar furnace for dental metallurgy will be described in another paper.

Any one familiar with the metallurgy of iron will see that the principles which have been used were discovered long ago. The hot blast was invented by Neilson in 1830. Siemens used gas as a fuel, and heated both this and the air. Using the waste gases to heat the blast is not new. Naphtha has been used to increase the illuminating power of gas, and knowing this, its value for increasing the heating power would occur to any one. Porous fire clay was known in Strabo's time, though T. Fletcher, with his usual capacity for adopting other men's ideas and giving them no credit, has recently reinvented it.

Though the principles were discovered long ago they had never been used together, and by this new combination I have been able to do what has never been done before in a gas furnace, produce heat enough to bake teeth, and this, too, in so short a time as forty minutes from the time the gas is lighted.

### CAUSES OF GASING IN FIRING MINERAL TEETH.

In my first experiments for making enamels for filling conspicuous cavities in teeth difficulty was sometimes found in making the enamel base of a pure translucent white. As the result of somewhat elaborate tests it was found that the gray or green tint was due to the reduction of some of the lead oxide. A similar difficulty was encountered when the enamel base was fritted with the metallic oxides necessary to give it the shade of the teeth. Here the oxides themselves were reduced.

It therefore seemed probable that the so-called gasing in mineral teeth came from a similar cause.

Many experiments and analyses were made to test the matter, and though it is not worth while to give these in detail, one experiment, which is easily tried, will be mentioned: After having made a furnace like the one described, light the gas and open the air valve. When the heat is high enough put an unbaked tooth into the muffle. When taken out the color will be pure. Now diminish the oxygen by partly closing the air valve. Put in another unbaked tooth. When this is removed it will be found gased. The same result will follow with the air valve in the same position as in the first experiment if the supply of gas is increased. In experiments with dental enamels and porcelains it is advisable to have a slight excess of air in the furnace. Where the color used, as, for example, platinum, yields the proper tint in the metallic state this precaution is unnecessary, as in these cases the color has no oxygen to give up.—*Boston Med. and Surg. Journal.*

